# An Unusual Association Between Metformin and Nightmares: A Case Report

**DOI:** 10.7759/cureus.28974

**Published:** 2022-09-09

**Authors:** Diana Voloshyna, Qudsia I Sandhu, Saima Khan, Anan Bseiso, Jaina Mengar, Nirupama Nayudu, Rajesh Kumar, Deepa Khemani, Muhammad Usama

**Affiliations:** 1 School of Medicine, University of Michigan, Ann Arbor, USA; 2 Medicine, Ghazi Khan Medical College, Dera Ghazi Khan, PAK; 3 Internal Medicine, Sir Syed College of Medical Sciences for Girls, Clifton, PAK; 4 College of Medicine, Al-Quds University, Jerusalem, PSE; 5 Medicine and Surgery, Government Medical College and New Civil Hospital, Surat, IND; 6 Internal Medicine, Zhengzhou University, Zhengzhou, CHN; 7 Medicine and Surgery, Jinnah Sindh Medical University, Karachi, PAK; 8 Neurology, Sheikh Zayed Medical College, Rahim Yar Khan, Rahim Yar Khan, PAK

**Keywords:** diabetes, bizzare and vivid dreams, abnormal dreams, metformin side effects, nightmares, metformin

## Abstract

Metformin remains the oral drug of choice for patients with type 2 diabetes mellitus (T2DM). It is an ideal anti-diabetic drug for maintaining good glycemic control in diabetics. However, the side effect profile of metformin varies from minor or no effects to substantial impact on the GI tract. In addition, metformin is rarely known for its association with nightmares. Here we present the case of a newly diagnosed 40-year-old diabetic who developed recurrent nightmares within a week of starting metformin treatment. The patient had no previous history of psychiatric or sleep disorders. However, it was the first time he had experienced such recurrent nightmares, especially after the start of 500 mg metformin thrice a day. Based on the Naranjo Adverse Drug Reaction (ADR) Probability Scale, and sudden onset and disappearance of nightmares after metformin initiation and discontinuation made metformin the primary cause of his nightmares.

## Introduction

Metformin's effectiveness, safety profile, cardiovascular and metabolic benefits, and ability to be combined with other anti-diabetic medications make it the preferred glucose-lowering therapy for patients with type 2 diabetes mellitus (T2DM) [1,2]. Metformin is primarily used to lower high blood sugar levels in patients with diabetes mellitus. It significantly improves overall glycemic control in patients with diabetes and promotes weight loss without causing low blood sugar [1]. However, metformin's side effect profile has caused concern among physicians worldwide. Based on nearly 60 years of research and clinical experience, metformin is generally considered safe, with the exception of nausea and gastroenteritis-related side effects and lactic acidosis [1,3]. However, a rare side effect of nightmares is reported infrequently in metformin patients [4]. Here we report a likely case of metformin-induced nightmares and abnormal dreams.

## Case presentation

A 40-year-old Asian male was recently diagnosed with T2DM and presented to the outpatient department with complaints of bizarre dreams occurring almost every night for the last three weeks. A month back, he visited his primary care physician, who diagnosed him with diabetes mellitus. His hemoglobin A1C (HbA1c) was 7.3% (4%-5.6% normal range), and his random blood sugar was 295 mg/dl. His labs, including his renal function tests (RFT), liver function tests (LFT), and lipid profile, were within the normal range. He is a nonsmoker and does not have hypertension. Consequently, he was started on a regular-release formulation of metformin 500 mg twice daily, along with dietary and lifestyle modification advice. The patient began having these bizarre nightmares within the first week of using metformin. As a school administrator, he initially attributed these weird dream occurrences to exhaustion and long work hours. However, the persistent nature of the nightmares after the start of metformin led him to suspect metformin as a root cause. He stated that it did not affect his sleep quality. Using the Pittsburgh Sleep Quality Index (PSQI), we determined that his sleep was of high quality. The patient is happily married and works in a wholesome atmosphere.

As he had never experienced stress or depression previously, he was concerned about the sudden and persistent character of his nightmares. The Hamilton Depression Rating Scale (HAMD-17) was negative for any depressive symptoms. In addition, the patient denied drug use and alcohol consumption.

His physical examination was unremarkable. His fundamental labs, including complete blood count (CBC), LFTs, and RFTS, were all normal (Table 1).

**Table 1:** Basic investigation report of the patient. Hb: Hemoglobin; MCV: Mean corpuscular volume; ALT: Alanine transaminase; AST: Aspartate transaminase; BUN: Blood urea nitrogen; Cr: Creatinine.

Complete blood count parameters	Value
Hb (g/dL)	13.8 (13.5-17.5)
MCV (fl)	88.8 (80-100)
WBC (X10^9^/l)	7.7 (4.5-11)
Platelets (X10^3^/ul)	350 (150-400)
ALT (IU/L)	24 (7-55)
AST (IU/L)	33 (8-48)
BUN (mg/dL)	14 (6-24)
Cr (mg/dL)	1.1 (0.7-1.3)
HbA1c%	7.1 (Below 5.7%)
Fasting blood glucose (mg/dL)	120 (99 or below)
Random blood glucose (mg/dL)	200 (140 or below)

His random blood glucose level was 200 mg/dl (normal: 140 mg/dL or below), and his fasting blood glucose level was 120 mg/dl (normal: 99 mg/dL or lower). He ruled out hypoglycemic attacks and dizziness. After taking metformin, he had nausea, but it subsided within a few hours. Upon reviewing his history and a detailed literature review, we identified metformin as the root cause of his symptoms. Therefore, on an experimental basis, we substituted glucagon-like peptide (GLP-1) agonist for metformin and instructed the patient to discontinue metformin use. Within the first week of discontinuing metformin, the patient denied having nightmares or unusual dreams. On his second follow-up at two weeks, there were still no nightmares. Two weeks later, on his second follow-up, he had not experienced any nightmares. To test our hypothesis, we decided to give the patient oral metformin, 500 mg twice a day, for a week to examine the effects on his dreams. On the second day of metformin treatment, the patient's nightmares returned, and they continued to occur nightly for as long as he remained on the medication. We further confirmed our diagnosis of metformin-induced nightmares by assessing Naranjo's Severity Assessment Scale and Hartwig's scale, which depicted a definitive causality and moderate association (Table 2) [5].

**Table 2:** Naranjo Adverse Drug Reaction Probability Scale showing a definitive cause based on the score of 9.

Naranjo Adverse Drug Reaction Probability Scale				
Questions	Yes	Nno	Do not know	Score
1. Are there previous conclusive reports on this reaction?	+1	0	0	1
2. Did the adverse event appear after the suspected drug was administered?	+2	-1	0	2
3. Did the adverse reaction improve when the drug was discontinued or a specific antagonist was administered?	+1	0	0	1
4. Did the adverse event reappear when the drug was re‐administered?	+2	-1	0	2
5. Are there alternative causes (other than the drug) that could on their own have caused the reaction?	-1	+2	0	2
6. Did the reaction reappear when a placebo was given?	-1	+1	0	+1
7. Was the drug detected in blood (or other fluids) in concentrations known to be toxic?	+1	0	0	0
8. Was the reaction more severe when the dose was increased or less severe when the dose was decreased?	+1	0	0	0
9. Did the patient have a similar reaction to the same or similar drugs in any previous exposure?	+1	0	0	0
10. Was the adverse event confirmed by any objective evidence?	+1	0	0	0
Total score :				9

## Discussion

Metformin, one of the oldest drugs in the biguanide class, is the first-line treatment for patients with T2DM [1]. It has gained superiority over other anti-diabetics because of its favorable metabolic and cardiovascular effects, efficacy, and high safety profile. In addition, recent in vitro and in vivo studies indicate that cancer patients exhibit antiproliferative activity [2].

Biguanides have been known to have a major adverse effect on diabetic patients. They elevate plasma lactate levels and lead to lactic acidosis in diabetic patients with additional risk factors such as chronic kidney disease, cirrhosis, hypoperfusion, or sepsis [3]. There is extensive research on the adverse effects of metformin, including lactic acidosis. However, there is less literature or case studies on other side effects like metformin-induced nightmares and sleep disturbance [4]. Sleep disturbance and insomnia are widespread complaints among patients who have T2DM, even in patients who do not take any anti-diabetic medication. Studies have been conducted to discover the prevalence of unsettling dreams and sleep disturbances in diabetic individuals. However, there is no detailed research on the causes of sleep disturbances and their association with diabetes. A validated questionnaire evaluated 614 individuals with type 2 diabetes, in which 48.2% experienced insomnia and 49% were poor sleepers, as shown by their PSQI score [6]. A cross-sectional study on 222 participants with childhood-onset type 1 diabetes showed that 26% of individuals had bad dreams, 41% of them had poor sleep quality, and 13% of participants experienced daytime sleepiness. The participants with poor glycemic control reported a greater frequency of bad dreams than patients with better glycemic control. If compared gender-wise, a greater proportion of female participants exhibited signs of poor sleep quality than male participants [7]. The above studies show sleep disturbances and nightmares due to diabetes mellitus. In contrast, diabetic patients undergoing drug therapy report sleep disorders, especially insomnia, and abnormal dreams after starting anti-diabetic medication. Metformin-induced insomnia is also prevalent among obese and elderly patients recently diagnosed with diabetes mellitus. The adverse effect of insomnia is more prevalent in patients than nightmares [4]. A case report talks about how a 56-year-old man started having nightmares and strange dreams after taking metformin. The adverse effect was graded on the Naranjo ADR scale. The patient's PSQI and HAMD-17 readings were normal, further excluding the possibility of depression or poor sleep quality. Nightmares may be caused by nocturnal hypoglycemia. However, metformin monotherapy does not produce hypoglycemia, particularly in the absence of fasting, malnutrition, vigorous exercise, and advanced age [8]. Hypoglycemia is uncommon in patients on metformin monotherapy, but Sulfonylureas carry a larger risk of hypoglycemia [2, 9]. The cerebral blood glucose level is connected to the body's dreaming mechanism. The diabetic individuals who experienced metformin-induced dreams had just recently begun taking metformin. The shift in blood sugar following the initiation of metformin therapy may be the sole conceivable explanation for the incidence of nightmares [4]. eHealthMe.com reported a 0.18% prevalence of metformin-induced nightmares among 417,750 people [10]. Metformin's side effects can be avoided by commencing pharmacological therapy with modest doses or by adopting an extended release release form [2].

## Conclusions

The relationship between metformin and nightmares is still the subject of much research. Sleep disturbances and insomnia are common concerns even among patients who do not take anti-diabetic medications. Insomnia is a more common adverse effect among patients than nightmares. The fundamental reason for sleep problems, bizarre dreams, and nightmares in metformin-treated individuals remains unknown. It may be attributed to a sudden imbalance in blood glucose levels upon the introduction of anti-diabetic medicine, resulting in hypoglycemia. However, the incidence of hypoglycemia with metformin is negligible. Abnormal dreams and nightmares are rare adverse effects of metformin in diabetic patients, and additional research is required to establish the link between metformin and nightmares.
